# Fanconi anaemia with bilateral diffuse pulmonary arterio venous fistulae: a case report

**DOI:** 10.1186/1471-2326-12-1

**Published:** 2012-03-17

**Authors:** Lasitha Samarakoon, Nuwan Ranawaka, Chaturaka Rodrigo, Godwin R Constantine, Lalindra Goonarathne

**Affiliations:** 1 University Medical Unit, University of Colombo, Colombo, Sri Lanka; 2 Department of Clinical Medicine, University of Colombo, Colombo, Sri Lanka; 3 Department of Pathology, Faculty of medicine, University of Colombo, Colombo, Sri Lanka

**Keywords:** Fanconi anaemia, Associations, Pulmonary arterio venous fistulae, Transthoracic echocardiogram, Contrast echocardiography, Right heart catheterisation

## Abstract

**Background:**

We report a patient with cytogenetically confirmed Fanconi anaemia with associated diffuse bilateral pulmonary arterio-venous fistulae. This is only the second reported case of diffuse pulmonary arterio-venous fistulae with Fanconi anaemia.

**Case Presentation:**

A 16 year old Sri Lankan boy, with a cytogenetically confirmed Fanconi anaemia was admitted to University Medical Unit, National Hospital of Sri Lanka for further assessment and treatment. Both central and peripheral cyanosis plus clubbing were noted on examination. The peripheral saturation was persistently low on room air and did not improve with supplementary Oxygen. Contrast echocardiography failed to demonstrate an intra cardiac shunt but showed early crossover of contrast, suggesting the possibility of pulmonary arterio-venous fistulae. Computed tomography pulmonary angiogram was inconclusive. Subsequent right heart catheterisation revealed bilateral diffuse arterio-venous fistulae not amenable for device closure or surgical intervention.

**Conclusion:**

To our knowledge, this is the second reported patient with diffuse pulmonary arterio-venous fistulae associated with Fanconi anaemia. We report this case to create awareness among clinicians regarding this elusive association. We recommend screening patients with Fanconi anaemia using contrast echocardiography at the time of assessment with transthoracic echocardiogram. Though universal screening may be impossible given the cost constraints, such screening should at least be performed in patients with clinical evidence of desaturation or when a therapeutic option such as haematopoietic stem cell transplantation is considered. Treatment of pulmonary arteriovenous fistulae would improve patient outcome as desaturation by shunting worsens the anaemic symptoms by reducing the oxygen carrying capacity of blood.

## Background

Fanconi anaemia (FA) is commonly inherited as an autosomal recessive trait but rarely can be an X-linked recessive disorder [1]. It is well known that FA patients display progressive bone marrow failure and an increased predisposition to malignancy [2]. They may also have one or more somatic abnormalities including dermatological (e.g. cafe'au lait spots), skeletal (e.g. hypoplastic thumbs, scoliosis), genitourinary (e.g. horse shoe kidney), gastrointestinal (e.g. duodenal atresia), cardiac and neurological abnormalities (VACTERL association). Approximately a third of FA patients have no obvious physical or somatic abnormalities. Although the majority of patients present towards the end of the first decade of life, it is increasingly noted that some patients with atypical presentations (e.g. myelodysplasia) are being first diagnosed in adulthood.

Due to improvements in medical care, many patients diagnosed in childhood are surviving into adulthood. We report the case of an adolescent male diagnosed with FA having bilateral diffuse pulmonary arterio venous fistulae.

## Case Presentation

A 16 year old Sri Lankan male, diagnosed with FA, presented to University Medical Unit (UMU) of National Hospital of Sri Lanka for further assessment of central cyanosis and clubbing. He was a product of a non consanguineous marriage and had been "healthy" up to 13 years of age when he had developed petichiae and found to have thrombocytopenia. There was no organomegaly. His platelet counts were persistently low around 20,000/ɥl. A trephine bone marrow biopsy showed severe bone marrow hypoplasia with haematopoietic tissue accounting for less than 25% of marrow spaces. No abnormal cells were noted and it was concluded to be compatible with severe aplastic anaemia. All other routine blood tests including liver function tests, renal function tests, erythrocyte sedimentation rate and tests of coagulation were within normal limits. Ultrasound scan of the abdomen was also normal. A clinical diagnosis of FA was made and genetically confirmed by chromosomal studies. Three chromosomal cultures with a) no mitomycin C (MMC), b) 10 ng/ml of MMC, c) 50 ng/ml of MMC all demonstrated increased chromosome breaks in metaphase chromosomal spreads in the patient when compared with a control. He continued to be followed up at a regional tertiary care centre where the progressive clubbing and cyanosis were noted. A transthoracic echocardiogram (TTE) performed initially by a paediatric cardiologist revealed a structurally and functionally normal heart without any evidence of an intra-cardiac shunt. Increased interstitial shadowing was noted in bilateral lung fields in the chest roentgenogram and lung function tests revealed a severe restrictive pattern. The high resolution CT (HRCT) of the thorax failed to demonstrate any evidence of pulmonary fibrosis.

Upon admission to UMU, in addition to central and peripheral cyanosis and grade 5 clubbing, the patient also had persistent desaturation with paltypnoea (breathlessness worsening on sitting up) and orthodeoxia (desaturation worsening with sitting up from lying down position). His oxygen saturation on pulse oximetry was persistently around 70% on air and further desaturation was noted upon standing.

A contrast echocardiography demonstrated early crossover of contrast during 3^rd ^and 5^th ^cycles raising the possibility of pulmonary arterio-venous fistula. No other cardiac cause was identified that could account for his cyanosis and clubbing. To account for the severe degree of desaturation, we assumed the shunt to be of large calibre. The CT pulmonary angiogram (CTPA) was inconclusive in this regard. A subsequent cardiac catheterisation study demonstrated bilateral diffuse pulmonary arterio-venous fistulae not amenable for surgical intervention.

## Discussion

FA is a genetically and phenotypically heterogeneous autosomal recessive disorder defined by cellular hypersensitivity to DNA cross-linking agents [3]. Clinically, FA is characterized by congenital malformations, progressive marrow failure, and predisposition to acute myelogenous leukemia (AML) and other malignancies [4].

Many different associations have been described in patients with FA. One is the VACTERL abnormality [5]. Complementation Group A Fanconi anaemia patient presenting with VACTERL association and atresia of the right ear has been reported [6]. FA with hydrocephalus and VACTERL have also been reported recently [7].

Multifocal neurological syndrome, concurrent thumb polydactyly and dorsal dimelia are other associations of FA [8,9]. Apart from the well known high incidence of haematological malignancies (particularly AML), FA is also associated with other malignancies [10]. Case reports of FA associated with squamous cell malignancies of the lower female genital tract [11,12], tongue [13,14], oral cavity [15] and in post cricoid area [16] have been reported. Both heterozygous and homozygous mutations in several FA predisposing genes were noted to be associated with an increased risk of squamous cell carcinoma of the oesophagus in a study in Iran [17]. Apart from squamous cell carcinomas, FA is also associated with liver tumours [18] and focal nodular hyperplasia of liver [19]. Other malignancies linked with FA include; Wilms' tumour, medulloblastoma [20] and retinoblastoma [21].

Regarding the pulmonary associations, a novel case of pulmonary glial heterotopia has been reported in association with FA and epilepsy [22]. Steens et al reported a case of pulmonary alveolar proteinosis with FA [23]. A single case of multiple pulmonary AV fistulae associated with Fanconi syndrome has been reported as far back as 1973 [24]. Despite an extensive literature survey we did not come across any further reported cases of FA associated with multiple pulmonary arterio venous fistulae making our case the second ever reported in medical literature during the span of nearly four decades. Whether this low incidence reflects chance association or non diagnosis due to lack of awareness and active screening is unclear. Cyanosis and clubbing in a patient with FA may be attributed to the coexistent cardiac anomalies (i.e. VACTERL) and active investigating might not be pursued with regard to confirming a pulmonary AV fistulae.

The reasons for these associations have not been clearly elucidated to date. Although the pathogenesis is obscure, abnormal cell migration has been suggested as a possible mechanism for pulmonary glial hetrotopia associated with FA [22]. Abnormal cytokine metabolism has been put forward as an explanation for the association of pulmonary alveolar proteinosis with FA [23]. Genetic mutations in genes associated with FA have been implicated as aetiological factors for development of malignancies [17]. It has been pointed out that all cases of FA, though genetically heterogeneous, will exhibit abnormalities in DNA repair. Though this may explain the predisposition to malignancies, it doesn't explain the other multiple structural abnormalities associated with FA [13].

Though FA patients with large arteriovenous malformations that become symptomatic are investigated and diagnosed, smaller asymptomatic arterio venous fistulae in others may pass unnoticed. Though asymptomatic, these malformations may cause significant desaturation with shunting and it will worsen the symptoms of anaemia by further reducing the Oxygen carrying capacity of blood. Even in the presence of an overt cardiac cause for cyanosis, we propose that it is prudent to screen for coexistent pulmonary AV fistulae as identifying and treating such AV fistulae may improve patient outcome. This is especially true in the context that such AV fistulae may complicate advanced treatment options for Fanconi anaemia such as haematopoietic stem cell transplantation. Less extensive arteriovenous fistulae may be amenable for minimally invasive device closure or even open surgical treatment.

We recommend transthoracic echocardiography (TTE) combined with contrast echocardiography as an initial screening tool for patients with evidence of desaturation to evaluate this elusive association of FA. Given the cost constraints if universal screening is not possible at least those who are considered for advanced therapeutic options such as stem cell transplant, or those showing clinical evidence of desaturation should undergo screening.

## Conclusions

FA is associated with haematological malignancies, solid organ malignancies and multiple somatic malformations for which a pathological explanation is yet incomplete. We report a very rare association with FA; extensive pulmonary AV fistulae causing desaturation and clubbing in a 16 year old patient. Detecting these malformations is of utmost importance as shunting of blood with resultant desaturation would worsen the symptoms of anaemia. Such AV fistulae may also complicate advanced treatment options such as haematopoietic stem cell transplantation. In this regard, we propose that at least those patients with FA with desaturation or awaiting advanced treatment options should undergo initial screening with a TTE combined with contrast echocardiography which is a cost effective screening tool available in many tertiary care centres.

## Consent

Written informed consent was obtained from the patient for publication of this case report and accompanying images. A copy of the written consent is available for review by the series editor of this journal.

## Abbreviations

FA: Fanconi anaemia; AML: Acute myeloid leukemia; AV fistula: Arterio venous fistula; UMU: University medical unit; TTE: Trans thoracic echocardiogram; CTPA: Computed tomography pulmonary angiogram; HRCT: High resolution computed tomography

## Competing interests

The authors declare that they have no competing interests.

## Authors' contributions

LBS gathered and analyzed the data and prepared the manuscript. NR and CR supervised the project, proofread and edited the final manuscript. GRC performed the contrast echocardiography and was responsible for overall cardiac care. LG was responsible for hematological aspects of management. Both GRC and LG supervised the project. All authors read and approved the manuscript.

## Pre-publication history

The pre-publication history for this paper can be accessed here:

http://www.biomedcentral.com/1471-2326/12/1/prepub
